# Supplementary material for: Evaluating diabetes care in primary healthcare centers in Abuja, Nigeria: a cross-sectional formative assessment
Source: BMC Prim Care. 2024 Jul 5;25:243. doi: 10.1186/s12875-024-02487-1 (PMC11227205; doi:10.1186/s12875-024-02487-1)
Supplement: Supplementary file 1 — Supplementary Material 1. [file 12875_2024_2487_MOESM1_ESM.docx]

APPENDIX A

Service Availability and Readiness Assessment (SARA): Adapted for Diabetes Integration formative assessment.

| Number Question Result | | |
| --- | --- | --- |
|  |  |  |
| 001 | Facility number |  |
| 001a | Date | Day month year |
| 001b | Interviewer Name |  |
| 003 | Name of facility |  |
| 004 | Location of facility |  |
| 005 | Region/Province (Council Area) |  |
| 006 | District (Ward) |  |
| 007 | Type of facility | HEALTH CENTRE/CLINIC.................................... 1  HEALTH POST........................................................ 2  OTHER (SPECIFY) ……………………………….. 3 |
| 008 | Managing Authority | GOVERNMENT/PUBLIC........................................... 1  NGO/NOT-FOR-PROFIT............................................ 2  PRIVATE-FOR-PROFIT............................................. 3  MISSION/FAITH-BASED.......................................... 4  OTHER (SPECIFY)…………………………………. 5 |
| 009 | Urban/Rural | URBAN ........................................................................ 1  RURAL ......................................................................... 2 |
| 010 | Outpatient only | YES .............................................................................. 1  NO................................................................................ 2 |
| 012 | Altitude | Meters |
| 013 | Latitude | N/S……........... a  DEGREES/DEC b – c |
| 014 | Longitude | E/W……........... a |
|  |  | DEGREES/DEC b  c |

Service Availability and Readiness Assessment (SARA): Adapted for Diabetes Integration formative assessment

| - Number Question Result Skip | | |
| --- | --- | --- |
| j7.8 INFORMED CONSENT (See Appendix B for Instructions) | | |
| **FACILITY NUMBER** INTERVIEWER CODE | | |
| **INTERVIEWER'S SIGNATURE INDICATING CONSENT OBTAINED DAY MONTH YEAR** | | |
| **015** | May I begin the interview? | YES...................................1  NO ...................................2 5001 |
| 016 | Interview start time  (see the 24 hour-clock system |  |
|  |  |  |

**SECTION 2: STAFFING**

| Indicator code | 200 | How many staff are currently assigned to, employed by, or are seconded to this facility? Count each staff member only once, on the basis of the highest technical or professional qualification. Of the total number, how many are part- time in this facility? | A  ASSIGNED/ EMPLOYED/  SECONDED (INCLUDING part time | B part yime |
| --- | --- | --- | --- | --- |
| S4 | 01` | Generalist (non-specialist) medical doctors |  |  |
| S4 | 02 | Specialist medical doctors |  |  |
| S4 | 03 | Non-physician clinicians/paramedical professionals |  |  |
| S4 | 04 | Nursing professionals |  |  |
|  | 08 | Pharmacists |  |  |
|  | 11 | Laboratory technicians (medical and pathology |  |  |
|  | 12 | Community health workers  |  |  |
|  | 13N | Community health officer |  |  |
|  | 14N | OTHER _________________________ (SPECIFY) |  |  |

Service Availability and Readiness Assessment (SARA): Adapted for Diabetes Integration formative assessment

| Indicator Number Question Result Skip  Code | | | | |
| --- | --- | --- | --- | --- |
| SECTION 3: PATIENT ACCESS AND CAPACITY | | | |  |
| **S2** | **301** | Excluding any delivery beds, how many overnight/inpatient beds in total does this facility have, both for adults and children? | # OF OVERNIGHT AND  INPATIENT BEDS: |  |
|  | **303N** | How do patients access the facility? | Walk-in only..................................... 1  Appointment only............................ 2  Combination of both ……………….. 3 |  |
|  | **304N** | Does the facility keep a record of patient visits? | Yes, for all visits............................... . 1  No __________________________ 2  SPECIFY) |  |
|  | **305N** | How are patient records kept? | Paper patient files.......................1  Electronic patient files.................2  Other_____________________ 3  (SPECIFY) |  |
|  | **306N** | What is the total number of visits to the health facility for outpatient services last month? | Total Number of  Visits Last Month |  |
|  | **307N** | This figure is based on | Register/Record .............................. 1  Estimation ....................................... 2 |  |
|  | **308N** | What is the total number of visits to the health facility for outpatient services yesterday? | Total Number of  Visits Yesterday |  |
|  | **309N** | This figure is based on | Register/Record .............................. 1  Estimation ....................................... 2 |  |
|  | **310N** | Of the patients who visited yesterday, how many visited for diabetes? | Total Number of Diabetes  Visits Yesterday: |  |

| **SECTION 4: NON-COMMUNICABLE DISEASES** | | | |
| --- | --- | --- | --- |
| 401a | Does this facility offer diagnosis of chronic diseases such as diabetes, cardiovascular disease, or chronic respiratory disease? | (0) No |  |
|  |  | (1) Yes |  |
| 401b | Does this facility offer management of chronic diseases such as diabetes, cardiovascular disease, or chronic respiratory disease? | (0) No |  |
|  |  | (1) Yes |  |
|  |  |  |  |
| ASK TO BE SHOWN THE LOCATION IN THE FACILITY WHERE NON-COMMUNICABLE DISEASE SERVICES ARE PROVIDED. FIND THE PERSON MOST KNOWLEDGEABLE ABOUT NCD SERVICES IN THE FACILITY, INTRODUCE YOURSELF, EXPLAIN THE PURPOSE OF THE SURVEY AND ASK THE FOLLOWING QUESTIONS. | | | |
| DIABETES | | | |
| 402 | Do providers in this facility diagnose and/or manage diabetes in patients? | (0) No |  |
|  |  | (1) Yes |  |
| Which of the following diabetes services does this facility provide? SELECT ALL THAT APPLY | | | |
| 403 | Diagnose diabetes | (0) No |  |
|  |  | (1) Yes |  |
|  |  | (2) Refer suspected case |  |
| 404 | Prescribe treatment for diabetes | (0) No |  |
|  |  | (1) Yes |  |
|  |  | (2) Refer suspected case |  |
| 405 | Provide follow up services for diabetic patients | (0) No |  |
|  |  | (1) Yes |  |
|  |  | (2) Refer suspected case |  |
| 406 | In what kind of setting is diabetes care provided at this facility? | (1)General Outpatient Clinic (Integrated) |  |
|  |  | (2)Specialist Diabetes Clinic |  |
|  |  | (3)Other__________________(specify) |  |
| Does this facility have any of the following documents in this service site?  IF YES, ASK: MAY I SEE THE DOCUMENT? | | | |
| 407 | A printed document with the national guidelines for the diagnosis and management of diabetes available in this facility today? | (0) No |  |
|  |  | (1) Yes, observed |  |
|  |  | (2) Yes, reported not seen |  |
| 408 | Any other guidelines for the diagnosis and management of diabetes available in this facility today? | (0) No |  |
|  |  | (1) Yes, observed |  |
|  |  | (2) Yes, reported not seen |  |
| 409 | A printed checklist or job aid for screening patients for risk of diabetes? | (0) No |  |
| 410 | A printed checklist or job aid that provides guidance for interventions for risk-based management of patients with risk factors for cardiovascular diseases and diabetes? | (1) Yes, observed |  |
|  |  | (2) Yes, reported not seen |  |
|  |  | (0) No |  |
| 411 | Cardiovascular risk assessment charts | (1) Yes, observed |  |
|  |  | (2) Yes, reported not seen |  |
|  |  | (0) No |  |
| 412 | Have you or any provider(s) of diabetes services received any training in the diagnosis and management of diabetes in the last two years? | (1) Yes, observed |  |
|  |  | (2) Yes, reported not seen |  |
|  |  | (0) No |  |
|  |  | (1) Yes |  |
| HEALTH MANAGEMENT INFORMATION SYSTEM FOR NCDS | | |  |
| 426 | Is there a register or database for patients diagnosed with NCDs where information such as patients’ treatment start date, clinic visit adherence, and outcomes are recorded? IF YES, ASK TO SEE THE REGISTER | (0) No |  |
|  |  | (1) Yes, a register documenting treatment start date, and outcomes is available |  |
|  |  | (2) Yes, a register documenting treatment start only is available |  |
| 426b | If yes, tick the NCDs applicable: | hypertension [ ], diabetes [ ], COPD [ ], SCD [ ]. |  |
| 427 | Does your clinic have a policy on using the patient registry for decision making about patient management? | (0) No |  |
|  |  | (1) Yes |  |
|  | How does the clinic use the information in the patient registry for decision making about patient management? [Select all that apply]  NOTE TO INTERVIEWER: Do not read out these options to the respondent | (0) Register not used for clinical decision making |  |
|  |  | (1) Generate patient reminders for clinic visits |  |
|  |  | (2)Identify patients who miss scheduled clinic visits |  |
|  |  | (3) Assess individual patient health outcomes e.g blood pressure control, blood glucose control |  |
|  |  | (4) Identify patient needs |  |
|  |  | (5)Assist clinicians in making individual patient treatment plans |  |
|  |  | (6)Monitor patient response to treatment |  |
|  |  | (7)Other_______________(specify) |  |
| 429 | Are NCD patient outcomes shared with facility staff through any means? | (0)Not shared |  |
|  |  | (1)Displayed in the facility |  |
|  |  | (2)Staff Meetings |  |
|  |  | (3)On individual basis as requested |  |
|  |  | (4)Other |  |
|  |  | (5)Do not know |  |
| 430 | Does the facility have an appointment system for routine follow-up for patients diagnosed with NCDs?IF YES, ASK TO SEE AN APPOINTMENT SCHEDULE FOR ANY NCD | (0) No |  |
|  |  | (1) Yes, schedule observed |  |
|  |  | (2)Yes, reported, not schedule seen |  |
| 431 | Are individual patient treatment cards maintained for patients with chronic diseases? IF YES, ASK TO SEE A PATIENT TREATMENT CARD | (0)No |  |
|  |  | (1)Yes, observed |  |
|  |  | (2)Yes, reported, not seen |  |
| 432 | Are individual patient records maintained for patients with NCDs? IF YES, CLARIFY THE TYPE OF RECORD AND ASK TO SEE EVIDENCE | (0)No |  |
|  |  | YES OBSERVED |  |
|  |  | (1) Paper patient records |  |
|  |  | (2) Electronic patient records |  |
|  |  | YES, REPORTED, NOT SEEN |  |
|  |  | (3) Paper patient records |  |
|  |  | (4)Electronic patient records |  |
| 433 | Do NCD patients at this clinic receive unique hospital numbers? | (0) No |  |
|  |  | (1) Yes, all patients |  |
|  |  | (2) Yes, some patients |  |
| 433 | Do patients use the same unique hospital number throughout all their interactions with different providers and departments such as pharmacy, laboratory, and inpatient and outpatient units? | (0) No |  |
|  |  | (1) Yes |  |
| NCD COUNSELLING SERVICES | | |  |
| NOW, I WILL LIKE TO KNOW ABOUT THE TYPES OF COUNSELING AND/OR HEALTH EDUCATION SERVICES YOUR HEALTH FACILITY PROVIDES TO PATIENTS AND THEIR FAMILIES ABOUT NON-COMMUNICABLE DISEASES. | | |  |
| 436 | Does this health facility have a list of topics routinely covered during your health talks | (0) No |  |
|  |  | (1) Yes |  |
| 437 | Which of the following counselling services are provided to patients about chronic diseases?[For each counselling topic offered, clarify if it is routinely offered to all patients, to those identified as at risk, or only on a case-by-case basis. Routinely means: the topics are included in the list of topics normally covered during health education and/or there is a written list of such topics available for inspection] | |  |
| 438 | Patient counselling and education on healthy diet [multiple options possible except when facility does not routinely offer counseling for NCDs] | (0) Not routinely offered in this facility |  |
|  |  | (1) Offered to all patients in the waiting area |  |
|  |  | (2) Offered to all patients identified at risk for chronic disease (e.g., aged 40 years or older; currently smoking, obese) |  |
|  |  | (3) Offered to patients with diagnosed chronic disease (e.g., hypertension, diabetes) |  |
| 439 | Patient counselling and education on smoking [multiple options possible except when facility does not routinely offer counseling for NCDs] | (0) Not offered to patients |  |
|  |  | (1) Offered to all patients in the waiting area |  |
|  |  | (2) Offered to all patients identified at risk for chronic disease (e.g., aged 40 years or older; currently smoking, obese) |  |
|  |  | (3) Offered to patients with diagnosed chronic disease (e.g., hypertension, diabetes) |  |
| 440 | Patient counselling and education on physical activity [multiple options possible except when facility does not routinely offer counseling for NCDs] | (0) Not offered to patients |  |
|  |  | (1) Offered to all patients in the waiting area |  |
|  |  | (2) Offered to all patients identified at risk for chronic disease (e.g., aged 40 years or older; currently smoking, obese) |  |
| 441 | Patient counselling and education on alcohol consumption [multiple options possible except when facility does not routinely offer counseling for NCDs] | (0) Not offered to patients |  |
|  |  | (1) Offered to all patients in the waiting area |  |
|  |  | (2) Offered to all patients identified at risk for chronic disease (e.g., aged 40 years or older; currently smoking, obese) |  |
|  |  | (3) Offered to patients with diagnosed chronic disease (e.g., hypertension, diabetes) |  |
| 442 | How is the counseling and education offered to patients with risk factors or who are already diagnosed with chronic disease? | (1`) In the waiting areas |  |
|  |  | (2) During consultation with a clinician |  |
|  |  | (3) During consultation with a counsellor |  |
|  |  | (4) Others____________ (Specify) |  |
| 443 | Does this health facility offer counselling and education of family members [of patients with chronic disease risk factors or established chronic disease] on smoking, diet, alcohol consumption, physical activity. | (0) No |  |
|  |  | (1) Yes |  |
| 444 | How is the counseling and education offered to family members of patients with chronic disease risk factors or established chronic disease? | (1) During consultation with a clinician |  |
|  |  | (2) During consultation with a counsellor |  |
|  |  | (3) By email/electronic materials |  |
|  |  | (4) Health coaching application |  |
|  |  | (5) Others____________ (Specify) |  |
| 445 | Does this health facility offer training or education for self-management | (0) No |  |
|  |  | (1) Yes for Hypertension |  |
|  |  | (2) Yes, for Diabetes |  |

**PATIENT HEALTH EDUCATION MATERIALS**

|  | Are there health education materials related to the following topics at this facility? | YES, observed | | Reported, not seen | | No |
| --- | --- | --- | --- | --- | --- | --- |
|  | **If YES, ask to see the materials and clarify if the materials are job aids for educating patients in the facility, materials to send home with the patient, or both.** | Job aids for patient education in facility | Materials for patient to take home | Job aids for patient education in facility | Materials for patient to take home |  |
| 446 | Smoking |  |  |  |  |  |
| 447 | Diet [Healthy Diet] |  |  |  |  |  |
| 448 | Alcohol consumption |  |  |  |  |  |
| 449 | Physical activity |  |  |  |  |  |
| 450 | Hypertension |  |  |  |  |  |
| 451 | Diabetes |  |  |  |  |  |

| **ADHERENCE MONITORING AND PATIENT TRACKING** | | | |
| --- | --- | --- | --- |
| 452 | What is your system for identifying hypertension and/or diabetes patients who miss their scheduled clinic visits? | (0) No system |  |
|  |  | (1) Review of patient registry |  |
|  |  | (2) Review of clinic appointment book |  |
|  |  | (3)Review of electronic appointment schedule |  |
|  |  | (4)Review of electronic medical records |  |
|  |  | (5)Others_________________(specify) |  |
| 453 | Is there a system for tracking patients with non-communicable diseases for follow up and adherence? | (0) No |  |
|  |  | (1) Yes |  |
| 454 | Does this hospital have a written standard operating procedure for tracking patients who miss clinic appointments? | (0) No |  |
|  |  | (1) Yes |  |
| 455 | Do you share tracking resources with any other program? | (0) No |  |
|  |  | (1) Yes, HIV Program |  |
|  |  | (2) Yes, MCH Program |  |
|  |  | (3) Yes, TB program |  |
|  |  | (4) Other________________(specify) |  |
|  | Indicate in the box whether the systems below are used to track chronic NCD patients who miss clinic appointment: Yes = 1; No = 0 | Hypertension | Diabetes |
| 456 | Trace through phone calls/text message |  |  |
| 457 | Trace through community volunteers |  |  |
| 458 | Tracking by facility focal point (person) |  |  |
| 459 | Treatment buddies |  |  |
| 460 | Appointment reminder systems |  |  |
| 461 | Other (Specify) |  |  |
| MEDICATION ADHERENCE SUPPORT | | | |
| 462 | Is there a system for promoting medication adherence among chronic NCD patients? | (0) No |  |
|  |  | (1) Yes |  |
| 463 | Indicate in the box whether the systems below are used to promote medication adherence among chronic NCD patients. Yes = 1; No = 0 | Hypertension | Diabetes |
| 464 | Adherence counseling |  |  |
| 465 | Treatment buddies |  |  |
| 466 | Pill counts |  |  |
| 467 | Text Message Reminders |  |  |
| 468 | Identifying late drug pick-up and follow-up |  |  |
| 469 | Health coaching |  |  |
| 470 | Other (Specify) |  |  |
| **PATIENT REFFERAL SYSTEM** | | | |
| 470a | Is there a protocol/guideline for deciding when to refer diabetes patients to a higher-level facility or a different provider? | (0) No  (1) Yes |  |
| 470 b | Is there a protocol/guideline for deciding when to refer hypertension patients to a higher-level facility or a different provider? | (0) No  (1) Yes |  |
| 471 | Is there a directory that lists the facilities/providers you refer patients with diabetes to? (If Yes, ask to see directory) | (0) No  (1) Yes, observed  (2) Yes, reported not seen |  |
| 472 | 471 b. Is there a directory that lists the facilities/providers you refer patients with hypertension to? (If Yes, ask to see directory) | (0) No  (1) Yes, observed  (2) Yes, reported not seen |  |
| 473 | Which of these method(s) are used to refer clients? [select all that apply] | (1) Verbal (tell them where to go)  (2) Issue standard referral form  (3) Blank paper to write referral information  (4) Telephone referral  (5) Escort client  (6) Other:………….. ……………….. (7) NA ______________________ |  |
| 474 | Is there a system for notifying the receiving facility/provider that a patient has been referred to them? | (0) No |  |
|  |  | (1) Yes |  |
| 475 | Is there a system to inform your facility that a client has completed the referral? | (0) No system |  |
|  |  | (1) Phone call |  |
|  |  | (2) Section of referral form filled out and sent back |  |
|  |  | (3) Separate counter-referral form |  |
|  |  | (4) Blank slip of paper |  |
|  |  | (5) Other_____________________ |  |
| 476  477 | Are patients ever referred back to this facility for follow-up after referral services are received? | (0) No |  |
|  |  | (1) Yes |  |
|  | Please describe the format in which you receive back referral information about patients sent back to your facility | (0) No information sent back |  |
|  |  | (1) Blank slip of paper |  |
|  |  | (2) Referral form |  |
|  |  | (3) Care summary form with test results |  |
|  |  | (4) Other________________(specify) |  |
| **EXTERNAL SUPERVISION FOR CHRONIC DISEASE SERVICE DELIVERY** | | | |
| 478 | When was the last time a supervisor from outside this facility came here on a supervisory visit related to any services for non-communicable diseases? Was it this month, within the past 2-3 months, or more than 3 months ago? | (0) No supervisory visit |  |
|  |  | (1) This month |  |
|  |  | (2) Within past 2-3 months |  |
|  |  | (3) More than 3 months ago |  |
|  |  | (4) I don’t know |  |
| 479 | Is there any documentation from an external supervisory visit for services for non-communicable diseases during the past 3 months?IF YES, ASK TO SEE DOCUMENTATION | (0)No |  |
|  |  | (1)Yes, Observed |  |
|  |  | (2)Yes, reported, not seen |  |
|  |  | (3) I don’t know |  |
| 480 | Does the documentation provide any feedback or comments on some aspect of management or services for non-communicable diseases?IF YES, ASK TO SEE DOCUMENTATION | (0)No |  |
|  |  | (1)Yes, Observed |  |
|  |  | (2)Yes, Reported, not seen |  |

EQUIPMENT AND COMMODITIES

| 481 | Please tell me if the following basic equipment and supplies used in the provision of client services are available anywhere in the outpatient service area and are functional. | (A) AVAILABLE | | | (B) FUNCTIONING | | |
| --- | --- | --- | --- | --- | --- | --- | --- |
|  |  |  | REPORTED,NOT SEEN | NOT AVAILABLE | YES | NO | DON’T KNOW |
| 482 | Adult weighing scale | 1 b | 2 b | 3 480 | 1 | 2 | 8 |
| 483 | Stethoscope | 1 b | 2 b | 3 481 | 1 | 2 | 8 |
| 484 | Height board/stadiometer | 1 b | 2 b | 3 482 | 1 | 2 | 8 |
| 485 | Measuring tape | 1 b | 2 b | 3 483 | 1 | 2 | 8 |
| 486 | Patella hammer | 1 b | 2 b | 3 484 | 1 | 2 | 8 |
| 487 | Ophthalmoscope | 1 b | 2 b | 3 485 | 1 | 2 | 8 |
| 488 | BMI Chart/Wheel | 1 b | 2 b | 3 486 | 1 | 2 | 8 |
| 489 | Monofilaments | 1 b | 2 b | 3 487 | 1 | 2 | 8 |
| 450 | Blood pressure apparatus (may be digital or manual sphygmomanometer with stethoscope) | 1 b | 2 b | 3 488 | 1 | 2 | 8 |
| 451 | How often are blood pressure measuring devices calibrated and checked for accuracy? | (0) Never |  |  |  |  |  |
|  |  | (1) Once a year or more |  |  |  |  |  |
|  |  | (2) Less than once a year |  |  |  |  |  |
|  |  | (3) I don’t know |  |  |  |  |  |
|  |  |  |  |  |  |  |  |

**THANK YOUR RESPONDENT AND MOVE TO YOUR NEXT DATA COLLECTION POINT IF DIFFERENT FROM CURRENT LOCATION**
