# Supplementary material for: Evaluating diabetes care in primary healthcare centers in Abuja, Nigeria: a cross-sectional formative assessment
Source: BMC Prim Care. 2024 Jul 5;25:243. doi: 10.1186/s12875-024-02487-1 (PMC11227205; doi:10.1186/s12875-024-02487-1)
Supplement: Supplementary file 2 — Supplementary Material 2. [file 12875_2024_2487_MOESM2_ESM.docx]

**APPENDIX B**

**DM INTEGRATION FORMATIVE STUDY @ 30 PHCs IN FCT, Abuja.**

**CHECKLIST FOR DIABETES SCREENING EQUIPMENT AND MEDICATION**

| **S/N** | **AREA COUNCIL** | **HEALTH FACILITY** | **ACs/HFs CODE** | **Functional glucometer observed.**  **Yes/No** | **Valid test strip observed.**  **Yes/No** | **A 30-day dose of metformin was observed.**  **Yes/No** | **A 30-day dose of Glibenclamide was observed.**  **Yes/No** |
| --- | --- | --- | --- | --- | --- | --- | --- |
|  |  |  |  |  |  |  |  |
|  |  |  |  |  |  |  |  |
|  |  |  |  |  |  |  |  |
|  |  |  |  |  |  |  |  |
|  |  |  |  |  |  |  |  |
|  |  |  |  |  |  |  |  |
|  |  |  |  |  |  |  |  |
|  |  |  |  |  |  |  |  |
|  |  |  |  |  |  |  |  |
|  |  |  |  |  |  |  |  |
|  |  |  |  |  |  |  |  |
|  |  |  |  |  |  |  |  |
|  |  |  |  |  |  |  |  |
|  |  |  |  |  |  |  |  |
|  |  |  |  |  |  |  |  |
|  |  |  |  |  |  |  |  |
|  |  |  |  |  |  |  |  |
|  |  |  |  |  |  |  |  |
|  |  |  |  |  |  |  |  |
|  |  |  |  |  |  |  |  |
|  |  |  |  |  |  |  |  |
|  |  |  |  |  |  |  |  |
|  |  |  |  |  |  |  |  |
|  |  |  |  |  |  |  |  |
|  |  |  |  |  |  |  |  |
|  |  |  |  |  |  |  |  |
|  |  |  |  |  |  |  |  |
|  |  |  |  |  |  |  |  |
|  |  |  |  |  |  |  |  |
|  |  |  |  |  |  |  |  |
